# Support Engineering Strategy to Tackle the Trade-Off Between Catalytic Reactivity and H_2_O_2_ Selectivity in Electro-Oxygen Reduction

**DOI:** 10.3390/ma19081594

**Published:** 2026-04-15

**Authors:** Zetao Song, Shuai Ran, Zengjian Cai, Yue Zhao, Xiaobing Yang, Zhe Wang, Guodong Sun, Yanan Cao, Li Li

**Affiliations:** 1 State Key Laboratory of Chemistry for NBC Hazards Protection, Beijing 102205, China; songzetao1998@outlook.com (Z.S.);; 2 School of Chemical Sciences, University of Chinese Academy of Sciences, Beijing 100049, China

**Keywords:** support engineering, electro-oxygen reduction, H_2_O_2_, ultrafine Pd nanoparticles

## Abstract

**Highlights:**

- Under the rotating ring-disk electrode conditions used in the study, co-doped supports markedly enhance 2e^−^ H_2_O_2_ selectivity.
- Tuning the support (rather than increasing Pd content) delivers higher H_2_O_2_ selectivity without increasing precious-metal usage.
- At comparable precious-metal loadings, prioritize N,S co-doped carbon supports to maximize 2e^−^ selectivity; S-doped supports offer a balanced alternative; N-doped supports are more suitable when the 4e^−^ pathway to water is desired.
- Desired selectivity is attainable at low Pd loading, favoring scale-up and cost control.

**Abstract:**

Direct electrochemical reduction of oxygen to hydrogen peroxide has garnered increasing research attention because of its mild and easy operation relative to the traditional anthra-quinone cycling route. However, currently used carbon supported noble metal electrocatalysts such as Pd and Pt in the form of single atoms or ultrafine nanoparticles greatly suffer from low reactivity and/or selectivity to hydrogen peroxide. We herein report that ultrafine ca. 1 nm Pd nanoparticles that are stabilized on a N and S co-functionalized car-bon support (Pd/NSC) display excellent reactivity and H_2_O_2_ selectivity toward electro-oxygen reduction reactions. Our support engineering strategy is expected to open up new opportunities to simultaneously attain high reactivity and H_2_O_2_ selectivity in electro-reductions of oxygen.

## 1. Introduction

Hydrogen peroxide (H_2_O_2_) is widely used in a variety of chemical industries and in environmental engineering, with an annual consumption of more than 4 million tons [1,2,3,4] Recently, direct 2e^−^ electro-reduction of oxygen to hydrogen peroxide under mild conditions has emerged as a promising alternative route to the traditional anthraquinone process carried out, which requires substantial energy input and involves complex hydrogenation and oxidation steps and entails complex hydrogenation and oxidation reaction procedures [5,6,7,8,9,10] To this end, developing highly efficient electrocatalysts that can promote electrochemical conversion of oxygen to hydrogen peroxide becomes of paramount importance.

Currently, carbon supported noble metals (e.g., Pd, Pt) in the form of single atoms or ultrafine nanoparticles (NPs) have been identified as efficient electrocatalysts to boost direct reduction of oxygen to hydrogen peroxide [11,12,13,14,15,16,17] In these systems, noble metal species are commonly dispersed and stabilized by heteroatoms such as sulfur, boron and phosphorus on the carbon skeleton [18], which endow the noble metal particles with an ultrafine size and abundant surface-exposed isolated atop sites that prove to be the active centers selectively converting oxygen to hydrogen peroxide [19,20] However, the activity of the ultrafine NPs of noble metals was still very low despite the excellent selectivity to hydrogen peroxide during the electro-oxygen reduction process.

Regarding theoretical insights into the electro-oxygen reduction reaction over the ultrafine noble metal NPs, theoretical studies have shown that oxygen was firstly adsorbed and activated on the surface of noble metals, forming the adsorbed HOO* species [21,22,23,24] The electrocatalytic activity and selectivity to hydrogen peroxide over the ultrafine noble metal NPs were intimately connected with the adsorption strength of the HOO* thereon. Strong HOO* binding would increase the reactivity while favoring O–O bond cleavage toward the 4e^−^-driven water formation process. In contrast, by weakening the HOO* adsorption strength, the 2e^−^-driven hydrogen peroxide formation would be largely favored at the cost of reducing overall reactivity for both the 2e^−^ and 4e^−^ electro-oxygen reduction reactions. As a consequence, how to simultaneously attain high activity and selectivity toward the 2e^−^ hydrogen peroxide formation reaction over the ultrafine noble metal electrocatalysts remains technically challenging [25] Recent studies have established that the 2e^−^ ORR pathway is highly sensitive to the adsorption strength and fate of the key HOO* intermediate; excessively strong interaction tends to promote O–O bond cleavage and drive the reaction toward the 4e^−^ pathway, whereas overly weak binding compromises catalytic activity [26] Therefore, an effective catalyst design should create a local catalytic environment that balances intermediate stabilization with facile desorption, thereby preserving the O–O bond while maintaining fast reaction kinetics.

In this context, heteroatom-functionalized carbon supports provide an attractive platform for tuning the geometric and electronic properties of Pd-based active sites. Recent studies [27] have shown that 2e^−^ ORR performance can be regulated by coordination/environmental atom engineering, while N,S-containing interfacial environments can further modulate the electronic state and dispersion of Pd species. These findings motivated us to explore N,S co-functionalized carbon as a Pd support to achieve a more favorable balance between activity and H_2_O_2_ selectivity.

Herein, we deliberately synthesized ultrafine Pd NPs (ca. 1 nm) that were supported on N and S co-functionalized carbon (Pd/NSC), serving as highly active and selective O_2_-to-H_2_O_2_ conversion electrocatalysts. Pd/NSC exhibited around 80% H_2_O_2_ selectivity over a wide potential window, with its activity (current density) displaying a five-fold increase in relation to that of the highly selective but less active Pd/SC at 0.45 V vs. a reversible hydrogen electrode (RHE, same hereafter). The N,S co-functionalized carbon support not only facilitates the formation of ultrasmall Pd NPs with a high fraction of atop-like surface sites that can facilitate electro-reduction of oxygen to hydrogen peroxide but also mediates the adsorption energy of HOO* on the surface of ultrafine Pd NPs to improve its reactivity. The support engineering method as we present here provides a new avenue to balance the reactivity and H_2_O_2_ selectivity for electro-oxygen reduction reactions.

## 2. Materials and Methods

### 2.1. Materials

All chemicals were of analytical grade and used as received without further purification. Na_2_PdCl_4_, urea, and sublimed sulfur were purchased from Sinopharm Chemical Reagent Beijing Co., Ltd. (Beijing, China). Orion printex U carbon was received as a gift from TICHEM (Shanghai, China). Deionized water with a resistivity of 18.2 MΩ-cm was obtained from a Milli-Q System and used in all experiments.

### 2.2. Preparation of N and/or S Functionalized Carbon Support

The N functionalized supports were prepared by a so-called “soft nitriding” method based on our previous report. In a typical synthesis, 2 g of printex U carbon powders were homogeneously mixed with 3 g urea and physically ground together. After which, the mixtures were transferred into a suitable crucible and fully sealed with aluminum foil. Subsequently, the crucible was subject to calcination at 150 °C for 2 h and 300 °C for another 4 h. The calcined products were then thoroughly washed with water to remove free N species of supports and dried in air at 60 °C overnight.

The NSC supports were prepared by solid-phase sulfurization of sublimated sulfur with N functionalized carbon supports (NC). In a typical synthesis, 1 g sublimed sulfur was homogeneously mixed with 0.2 g NC and physically ground together. After which, the mixtures were transferred into a suitable crucible and fully sealed with aluminum foil. Subsequently, the crucible was subject to calcination at 400 °C for 1 h under an Ar flow atmosphere. After cooling to room temperature, the excessive sulfur was removed by hot KOH (2 M, 60 °C) etching. By varying the weight ratio of NC to sublimed sulfur, three NSC supports with varied N:S ratios were synthesized. The preparation of the S functionalized carbon support was similar to the preparation of NSC except that when synthesizing SC, Orion printex U carbon powder is used instead of the NC support.

### 2.3. Preparation of Ultrafine Pd NPs on N and/or S Functionalized Carbon Supports

Synthesis of ultrafine Pd NPs on N and/or S functionalized carbon supports was carried out by an impregnation method. Typically, 47 μL of 0.1 M Na_2_PdCl_4_ solution was added to 20 mL of deionized water, and 50 mg of NSC supports were added subsequently. After stirring for 12 h, the suspension was thoroughly washed by centrifugation. The products were dried overnight at 60 °C and then reduced with hydrogen at 300 °C for 2 h. Taking a similar preparation, the Pd/NC, Pd/SC and Pd/C catalysts were prepared as controls accordingly.

### 2.4. Characterizations

The powder X-ray diffraction (XRD) measurements were carried out on a Rigaku Smart Lab (Rigaku Corporation, Tokyo, Japan) powder diffractometer using Cu Kα radiation (λ = 0.154 nm, 9 kW). The X-ray photoelectron spectroscopy (XPS) characterizations were carried out on a Thermo ESCA-LAB 250Xi (Thermo Fisher Scientific Inc., Waltham, MA, USA). The measured binding energies were corrected by referencing the C 1s peak of adventitious carbon to 284.5 eV. Quantitative analysis of the chemical composition was carried out using an Agilent ICP-OES 730 (Agilent Technologies, Santa Clara, CA, USA). High-resolution transmission electron micros-copy (HR-TEM) images and EDS line scans were conducted on a Talos F200X (Thermo Fisher Scientific Inc., Waltham, MA, USA) microscope with an energy dispersive spectrometer.

### 2.5. Electrochemical Reduction of O_2_ to H_2_O_2_ Evaluations

The catalyst ink was firstly made as follows: 2.45 mg of the catalyst was dispersed in a mixed solution of isopropanol (750 μL), water (240 μL) and Nafion (10 μL, AlfaAcesar, Ward Hill, MA, USA, 5wt %). The suspension was then treated by sonication for 30 min to obtain a uniformly dispersed ink. The CHI760 electrochemical workstation was employed to record the electrochemical performance. A typical three-electrode system was used in the electrochemical tests. Graphite electrode and saturated calomel electrode (SCE) were used as counter electrode and reference electrode respectively. A rotating ring-disk electrode (RRDE) consisting of a glassy carbon disk electrode (Φ = 5.61 mm, as a catalyst support) and a Pt ring (Φ = 7.92 mm) was the working electrode in the test. To prepare the working electrode, 10 µL of the catalyst ink was drop-casted onto the disk electrode of the RRDE. The reference electrode was calibrated by the equilibrium potential of HER/HOR on the Pt/C electrode under a H_2_-saturated atmosphere. The catalyst evaluations were conducted in the electrolyte of 0.2 M PBS (pH = 7.0) at room temperature under the O_2_-saturated atmosphere, and the rotation speed was set as 1600 rpm. The catalyst was activated to a stable state by several cycles of the cyclic voltametric (CV) scanning with a sweep rate of 50 mV s^−1^, and a stable polarization curve was obtained by the linear sweep voltammetry (LSV) with a slow sweep rate of 5 mV s^−1^ from 0.2 to 0.8 vs. RHE. In the test, the applied voltage of the Pt ring was set to be 1.2 V (vs. RHE). The two current curves obtained in the experiment correspond to the disk current (i_D_) and the ring current (i_R_). The peroxide selectivity is calculated using the formula as follows:

(1) $$H_{2}O_{2}=200\times \frac{i_{R}/N}{\left|i_{D}\right|+i_{R}/N}$$

where i_R_ is the ring current, i_D_ is the disk current, and N is the collection efficiency of the ring-disk electrode. In this study, the collection efficiency was calibrated to 36.7% using the ferrocyanide/ferricyanide half-reaction system.

## 3. Results and Discussion

### 3.1. Preparation of Heteroatom-Functionalized Supports and Structural Features of the Pd Catalysts

We have prepared ultrafine Pd NPs on N and S co-functionalized carbon supports using a surface-confinement method we developed recently [28] To begin with, a nitrided carbon (NC) support was made by 300 °C thermal annealing of the mixture of physically ground urea and Printex U carbon in air. The NC support was then allowed for S-functionalization via a 400 °C thermal treatment of the homogeneously mixed NC support and sublimed sulfur under argon. After which, the N and S co-functionalized carbon supports were obtained. By varying the weight ratio of NC to sublimed sulfur, three NSC supports with varied N:S ratios of 2.7:1 (NSC1), 2.5:1 (NSC2), and 2.3:1 (NSC3) were synthesized. The energy-dispersive X-ray spectrometry (EDS) elementary mapping images of the NSC1, NSC2, and NSC3 supports in Figure S1 display that N and S elements are homogeneously dispersed throughout the carbon framework, indicating successful N and S co-functionalization of carbon support. As a control, pure N-functionalized carbon supports (NC) and pure S-functionalized carbon supports (SC) were also prepared. To improve the clarity of the sample description, Table 1 summarizes all supports and Pd-based catalysts investigated in this work, including their functionalization, N:S ratio, Pd loading, particle size, and role in the study.

**Figure 1:**
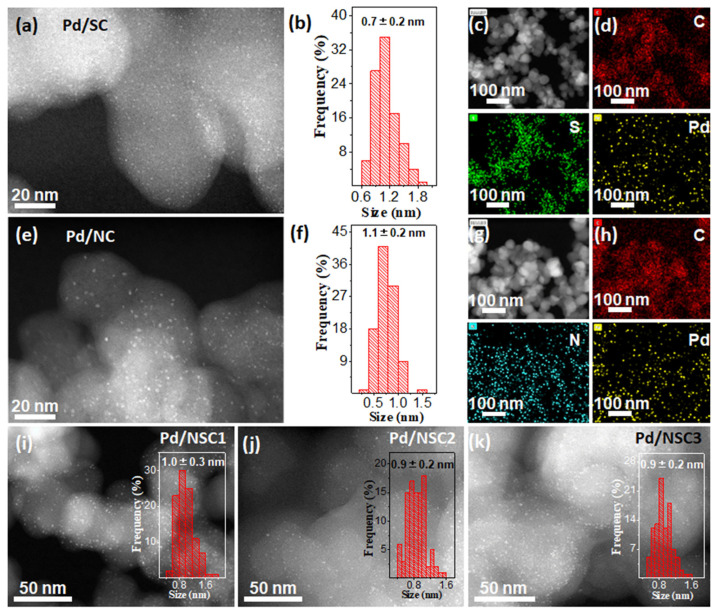
(**a**,**c**) HAADF-STEM images of Pd/SC with varied magnifications. (**d**) EDS mapping images of Pd/SC. (**e**,**g**) HAADF-STEM images of Pd/NC with varied magnifications. (**h**) EDS mapping images of Pd/NC.( In the elemental mapping images, red, green, cyan, and yellow correspond to C, S, N, and Pd, respectively.) (**i**) HAADF-STEM image of Pd/NSC1. (**j**) HAADF-STEM image of Pd/NSC2. (**k**) HAADF-STEM image of Pd/NSC3. (**b**,**f**) and the insets of (**i**,**j**,**k**) are corresponding histograms of particle-size distribution of Pd/SC, Pd/NC, Pd/NSC1, Pd/NSC2, and Pd/NSC3, respectively.

Pd^2+^ cations were subsequently adsorbed onto the NC, SC, and NSC supports by virtue of aqueous impregnation (see Supplementary Materials). After 300 °C hydrogen reduction, the N and/or S stabilized-Pd^2+^ cations were in situ reduced to ultrafine Pd NPs on supports. As shown in Figure 1 and Figure S2, the synthesized Pd NPs on a NC support (Pd/NC), SC support (Pd/SC), and NSC supports (Pd/NSC1, Pd/NSC2, Pd/NSC3) are measured to be around 1 nm, with a narrow size distribution, as given by the high-angle annular dark-field scanning transmission electron microscopy (HAADF-STEM) observation. EDS elementary mapping analysis of Pd/NC, Pd/SC and Pd/NSC in Figure 1d,h and Figure S3 discloses that the Pd element distribution is in good agreement with that of the N and/or S elements, implying the critical role of N and/or S in dispersing and stabilizing the synthesized ultrafine Pd NPs thereon. By comparison, Pd NPs that were prepared on a pure carbon support without N and/or S functionalization displayed a very broad size distribution, with an average particle size of 4.6 ± 5.3 nm, as presented in Figure S4, which revealed the great importance of N and/or S functionalization to make ultrafine Pd NPs. It is worth noting that the average Pd particle sizes in Pd/NC, Pd/SC, Pd/NSC1, Pd/NSC2, and Pd/NSC3 are all close to that of Pd10 nanoclusters (ca. 1 nm), which is expected to provide a high proportion of atop-like Pd sites favorable for the two-electron oxygen reduction pathway toward H_2_O_2_ formation.

**Figure 2:**
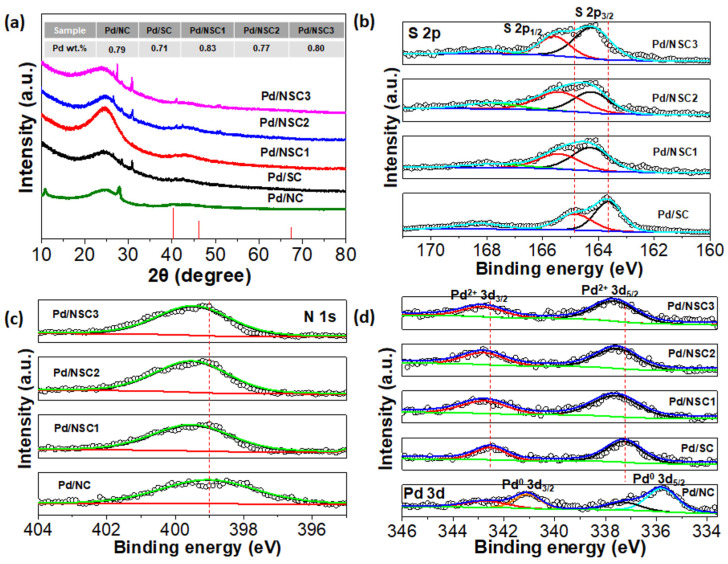
(**a**) XRD patterns of Pd/NC, Pd/SC, Pd/NSC1, Pd/NSC2, and Pd/NSC3. The vertical lines represent the standard peaks for Pd (PDF no. 46-1043) (**b**) S 2p XPS spectra of Pd/SC, Pd/NSC1, Pd/NSC2, and Pd/NSC3. (**c**) N 1s XPS spectra of Pd/NC, Pd/NSC1, Pd/NSC2, and Pd/NSC3. (**d**) Pd 3d XPS spectra of Pd/NC, Pd/SC, Pd/NSC1, Pd/NSC2, and Pd/NSC3. The inset of (**a**) is Pd weight loadings of Pd/NC, Pd/SC, Pd/NSC1, Pd/NSC2, and Pd/NSC3 as given by the ICP-OES measurements.

The ultrafine particle size of Pd NPs in Pd/NC, Pd/SC, and Pd/NSC is also evidenced by the wide-angle X-ray diffraction (XRD) spectra in Figure 2a, in which the characteristic XRD reflection peaks for Pd (PDF no. 46-1043) are too broad to be identified in spite of their relatively high Pd weight loadings (about 0.8wt.%), which are determined by the inductively coupled plasma optical emission spectrometer (ICP-OES) measurements, as shown in the inset of Figure 2a. The Pd weight loadings of Pd/NC, Pd/SC, Pd/NSC1, Pd/NSC2, and Pd/NSC3 are measured to be 0.79wt.%, 0.71wt.%, 0.83wt.%, 0.77wt.%, and 0.8wt.%, respectively. By comparison, the Pd/C sample prepared using the same procedure exhibits a Pd loading of 0.86 wt.% according to ICP-OES and shows well-defined Pd diffraction peaks in Figure S5, consistent with the much larger Pd particles observed in Figure S4. Moreover, Pd/C displays a markedly lower H_2_O_2_ selectivity than Pd/NC, Pd/SC, and the Pd/NSC catalysts, further confirming that heteroatom-functionalized supports are not merely passive carriers but actively regulate Pd nucleation and growth to generate ultrasmall Pd structures favorable for selective H_2_O_2_ electrosynthesis.

### 3.2. Electronic Interactions Among N, S, and Pd

Figure 2b demonstrates the S 2p X-ray photoelectron spectroscopy (XPS) spectra of Pd/SC, Pd/NSC1, Pd/NSC2, and Pd/NSC3. It was found that the oxidation state of the sulfur species in Pd/SC, Pd/NSC1, Pd/NSC2, and Pd/NSC3 are S^2−^ [22] Compared with the binding energy peaks of the S^2−^ in Pd/SC, the S^2−^ binding energy peak position of Pd/NSC1, Pd/NSC2, and Pd/NSC3 displayed an obvious positive shift. To make it clear, the S^2−^ 2p_3/2_ and S^2−^ 2p_1/2_ binding energy peaks of Pd/SC are centered at 163.7 eV and 165 eV, respectively, while those of Pd/NSC1, Pd/NSC2, and Pd/NSC3 are around 164.3 eV and 165.6 eV, indicating about a 0.6 eV positive shift. A similar trend was also identified in the N 1s XPS spectra of Pd/NC, Pd/NSC1, Pd/NSC2, and Pd/NSC3 in Figure 2c, where the N 1s binding energies of the Pd/NSC catalysts are shifted relative to that of Pd/NC. The S 2p and N 1s binding energy increase of Pd/NSC1, Pd/NSC2, and Pd/NSC3 relative to that of Pd/SC and Pd/NC revealed strong cooperativity between N and S in Pd/NSC, which possibly derived from the initial state effects of the XPS connecting with the ground state valence charge of the photoexcited atoms [29,30] Specifically, the electronegativity of the N (3.04) element was much larger than the S (2.58) element [31] which greatly facilitated electron transfer from S to N in the NSC support and accordingly made Pd/NSC display a higher binding energy of S^2−^ in comparison with that of Pd/SC. In another respect, the negatively charged S^2−^ would impede photo-excitation of the N 1s inner shell electron because of the electrostatic repulsion, hence largely favoring the positive shift of binding energy peaks of N 1s of Pd/NSC relative to that of Pd/NC. Therefore, the NSC support should not be regarded simply as a physical combination of NC and SC; rather, the co-functionalization creates a new local electronic environment that can influence the adsorption and reduction behavior of Pd precursors and, ultimately, the electronic structure of the supported Pd nanoparticles.

The N and S synergies of the NSC support have a great influence on the oxidation state of ultrafine Pd NPs thereon. For example, due to the ultrafine size of Pd NPs (0.7 ± 0.2 nm, Figure 1b) of Pd/SC, the Pd species thereon are well attributed to Pd^2+^, as displayed by the Pd 3d XPS spectra in Figure 2d, which is in good agreement with previous reports for ultrafine noble metal NPs [32,33] Meanwhile, the Pd 3d_3/2_ and Pd 3d_5/2_ binding energies of Pd/NSC present a ca. 0.4 eV increase compared with that of Pd/SC, resulting from the synergies between N and S. To be noted, both Pd^0^ and Pd^2+^ species were identified for Pd/NC, as shown in Figure 2d, as a result of its relatively larger Pd NPs (1.1 ± 0.2 nm, Figure 1f), which was also identified in a previous report [33]

Taken together, these XPS results indicate that the N,S co-functionalized support should not be regarded as a simple physical combination of NC and SC. Instead, the coexistence of N and S creates a distinct local electronic environment through their cooperative interaction, which perturbs the electronic structure of the supported Pd nanoparticles. In this sense, the role of N,S co-functionalization is synergistic in nature: it not only stabilizes ultrasmall Pd species on the carbon support but also modifies the local charge distribution around Pd, thereby providing the basis for tuning ORR activity and selectivity simultaneously.

### 3.3. Electrocatalytic Performance and Structure–Performance Relationship

**Figure 3:**
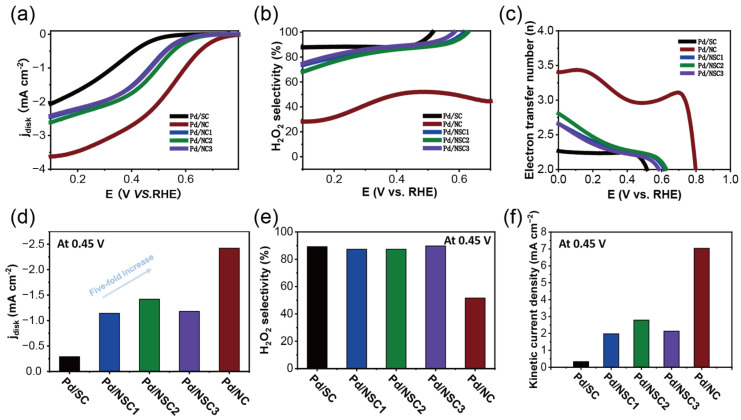
Electrocatalysis evaluations for the oxygen reduction reaction over the Pd/NC, Pd/SC, Pd/NSC1, Pd/NSC2, and Pd/NSC3 electrocatalysts in a neutral electrolyte (0.2 M phosphate-buffered saline, PBS, pH ≈ 7). (**a**) Linear sweep voltammetry (LSV) and (**b**) H_2_O_2_ selectivity curves of Pd/NC, Pd/SC, Pd/NSC1, Pd/NSC2, and Pd/NSC3. (**c**) Electron transfer number (n). (**d**) Current density and (**e**) H2O2 selectivity of Pd/NC, Pd/SC, Pd/NSC1, Pd/NSC2, and Pd/NSC3 at 0.45 V (RHE). (**f**) Kinetic current density (j_k_) of Pd/SC, Pd/NC, and Pd/NSC catalysts at 0.45 V(RHE).

The electrocatalytic oxygen reduction reaction (ORR) performance of the catalysts was evaluated in an O^2−^ saturated neutral electrolyte by RRDE measurements. As shown in Figure 3a, Pd/NC exhibits a substantially higher current density than Pd/SC over the tested potential range, indicating a higher ORR activity. However, this increase in activity is accompanied by a pronounced loss in H_2_O_2_ selectivity (Figure 3b). In contrast, Pd/SC shows much lower activity but maintains a markedly higher H_2_O_2_ selectivity across a wide potential window. This comparison highlights the typical trade-off between activity and selectivity in the two-electron ORR; a catalyst that activates oxygen more readily does not necessarily favor H_2_O_2_ as the final product.

In order to better compare the ORR kinetics of these catalysts, Tafel plots were constructed from the polarization data, as shown in Figure S8. The apparent Tafel slope of Pd/SC is 33 mV dec^−1^, which is smaller than those of Pd/NC (59 mV dec^−1^) and the N,S co-functionalized catalysts (47–50 mV dec^−1^), indicating clear support-dependent differences in ORR kinetics. Notably, although Pd/SC exhibits the smallest Tafel slope, its overall current density remains much lower than that of Pd/NC, whereas the N,S co-functionalized catalysts display intermediate kinetic behavior. Pd/NSC2 provides a better balance between kinetics and H_2_O_2_ selectivity. These results suggest that the role of N,S co-functionalization is not simply to maximize ORR kinetics, but rather to place Pd in a more favorable activity–selectivity regime.

A representative potential of 0.45 V vs. RHE was selected because it lies in the intermediate ORR region, where the catalysts still deliver measurable current densities while retaining distinct differences in H_2_O_2_ selectivity, thus allowing a meaningful comparison of the activity–selectivity balance. The average electron transfer number (*n*) at 0.45 V was estimated from the RRDE-derived H_2_O_2_ selectivity according to *n* = 4 − H_2_O_2_(%)/50. The calculated *n* values are 2.21 for Pd/SC, 2.97 for Pd/NC, 2.25 for Pd/NSC1, 2.25 for Pd/NSC2, and 2.21 for Pd/NSC3, indicating that the ORR on Pd/SC and Pd/NSC catalysts predominantly proceeds through the two-electron pathway, whereas Pd/NC shows a substantially stronger tendency toward the four-electron route.

To further evaluate the intrinsic ORR kinetics, the kinetic current density (j_k_) was calculated using the Koutecky–Levich equation after correcting for mass-transport limitation. At 0.45 V vs. RHE, the calculated j_k_ values are 0.33, 1.98, 2.79, 2.13, and 7.04 mA cm^−2^ for Pd/SC, Pd/NSC1, Pd/NSC2, Pd/NSC3, and Pd/NC, respectively. These results show that the N,S co-functionalized catalysts exhibit substantially higher intrinsic ORR kinetics than Pd/SC. Among them, Pd/NSC2 gives the highest j_k_ within the Pd/NSC series, indicating the most favorable kinetic behavior among the co-functionalized samples. Although Pd/NC exhibits the largest j_k_, its much larger *n* value and significantly lower H_2_O_2_ selectivity indicate that this higher activity is accompanied by a stronger tendency toward the four-electron pathway. Therefore, Pd/NSC2 achieves a more favorable balance between intrinsic ORR kinetics and H_2_O_2_ selectivity.

A more detailed analysis for the electronic structure with catalytic behavior was therefore carried out; the Pd 3d_5/2_ binding energy was correlated with the H_2_O_2_ selectivity and kinetic current density at 0.45 V vs. RHE (Figure S9). Although the relationship is not strictly monotonic, the correlation clearly shows that catalysts with a more favorable Pd electronic state exhibit a better balance between intrinsic ORR kinetics and preservation of the two-electron pathway. In particular, Pd/NC delivers the highest j_k_ but also a much larger *n* value and lower H_2_O_2_ selectivity, indicating a stronger tendency toward the four-electron pathway. In contrast, Pd/SC retains a pathway close to the 2e^−^ ORR route but suffers from much lower kinetic current density. Among the co-functionalized catalysts, Pd/NSC2 combines a shifted Pd 3d_5/2_ binding energy with the highest j_k_ in the Pd/NSC series while maintaining an *n* value close to 2, suggesting a more favorable electronic window for balancing ORR activity and H_2_O_2_ selectivity.

The behavior of Pd/C further supports the importance of structural control. As shown in Figure S6, Pd/C delivers an even lower H_2_O_2_ selectivity than Pd/NC. This result can be attributed to its much larger Pd particles (4.6 ± 5.3 nm), which are expected to expose a higher proportion of contiguous Pd ensembles and three-fold hollow sites. Such sites facilitate stronger O–O bond activation and cleavage, thereby favoring the four-electron pathway to water rather than the two-electron pathway to H_2_O_2_ [19] In contrast, the ultrasmall Pd particles in Pd/NC, Pd/SC, and Pd/NSC provide a much larger fraction of low-coordination atop-like sites, which are more compatible with selective HOO* formation and release.

Most importantly, the N,S co-functionalized catalysts successfully bridge the gap between Pd/NC and Pd/SC. As shown in Figure 3a–e, all Pd/NSC catalysts exhibit substantially higher current densities than Pd/SC while preserving a similarly high H_2_O_2_ selectivity of about 90%. For example, Pd/NSC2 delivers an approximately five-fold higher current density than Pd/SC at 0.45 V, while retaining nearly the same H_2_O_2_ selectivity. At the same potential, its activity is already comparable to that of Pd/NC, but its selectivity remains far superior. These results clearly demonstrate that N,S co-functionalization is able to break the conventional activity–selectivity compromise to a considerable extent.

The superior performance of Pd/NSC can be understood as a synergistic consequence of geometric and electronic regulation induced by N,S co-functionalization. From the geometric perspective, the co-functionalized support stabilizes Pd in an ultrasmall size regime, which maximizes the fraction of low-coordination Pd sites favorable for the two-electron ORR pathway. This geometric effect is reflected by the clear difference between Pd/C and the heteroatom-functionalized catalysts. From the electronic perspective, the support-induced shifts in the S 2p, N 1s, and Pd 3d binding energies indicate that N and S act cooperatively to generate a distinct local electronic environment around Pd, rather than behaving as two independent functionalities. Such electronic perturbation is consistent with moderated adsorption of ORR intermediates, likely including HOO*, which helps enhance oxygen activation relative to Pd/SC while suppressing excessive O–O bond cleavage relative to Pd/NC. Therefore, the improvement in Pd/NSC does not arise from a single factor, but from the synergy between structural stabilization and electronic-state modulation.

The comparison between Pd/NSC1, Pd/NSC2, and Pd/NSC3 further suggests that the synergy between N and S is composition-dependent. Although all three co-functionalized catalysts outperform Pd/SC in activity while maintaining high H_2_O_2_ selectivity, Pd/NSC2 exhibits the most favorable overall balance. This result implies that the cooperative effect of N and S is not simply proportional to the heteroatom content, but depends on an appropriate N:S ratio that optimizes the interplay between Pd stabilization, local electronic perturbation, and ORR intermediate regulation. Excessive deviation from this balance may weaken either the activity enhancement or the selectivity preservation, even when the Pd particle size remains in the ultrasmall regime.

Recent Pd-based studies further support the importance of local-structure engineering in H_2_O_2_ electrosynthesis. Pentagonal layered PdSe_2_ has been shown to deliver rapid H_2_O_2_ production in buffered neutral solution under high current density [34], while Pd sub-nanoclusters on NiTe_2_ achieved very high H_2_O_2_ selectivity through accelerated proton-coupled electron transfer [35] Compared with these representative Pd-based catalysts, our Pd/NSC system represents a different support-engineering route, in which N,S co-functionalized carbon regulates both the geometric dispersion and the electronic state of ultrasmall Pd nanoparticles through a synergistic support effect. The resulting catalyst does not simply maximize a single metric, but instead achieves a favorable balance between ORR kinetics and H_2_O_2_ selectivity in a neutral electrolyte.

### 3.4. Stability of the Pd/NSC Catalyst

The durability of the co-functionalized catalyst was examined using Pd/NSC2 as the representative sample. As shown in Figure S7, the current density remains largely stable during a 12 h test at 0.5 V, indicating that the catalyst maintains its ORR activity under continuous operation. The H_2_O_2_ selectivity shows only a slight decrease over time. According to recent reports, this behavior may originate, at least in part, from anion poisoning at the ring electrode rather than from severe degradation of the catalyst itself [36] Therefore, the stability test suggests that the Pd/NSC catalyst possesses reasonably robust catalytic properties in a neutral electrolyte, further supporting the practical potential of the N,S co-functionalization strategy for selective H_2_O_2_ electrosynthesis.

## 4. Conclusions

To summarize, we report a support engineering strategy to largely promote the conversion efficiency of oxygen to hydrogen peroxide during electro-reduction of oxygen reaction. A model Pd/NSC catalyst was carefully built by stabilizing ultrafine Pd NPs on a N and S co-functionalized carbon support. Pd/NSC displayed much enhanced reactivity while retaining similar H_2_O_2_ selectivity to the highly selective but less active Pd/SC catalysts. The improved reactivity–selectivity balance of Pd/NSC likely originates from its combined geometric and electronic regulation induced by N,S co-functionalization, which places Pd in a more favorable interfacial environment for balancing ORR kinetics and preservation of the two-electron pathway toward H_2_O_2_ induced by N,S co-functionalization. Our support engineering strategy likely opens up new opportunities to tackle the trade-off between catalytic reactivity and H_2_O_2_ selectivity for electro-reduction of oxygen.

## Figures and Tables

**Table 1:** Summary of the tested supports and Pd catalysts used in this study.

Sample	Type	Support Functionalization	N:S Ratio	Pd Loading (wt.%)	Average Pd Size (nm)	Role in Study
C	Support	-	-	-	-	Bare carbon control
NC	Support	N-functionalized carbon	-	-	-	Support control
SC	Support	S-functionalized carbon	-	-	-	Support control
NSC1	Support	N,S co-functionalized carbon	2.7:1	-	-	Co-functionalized support
NSC2	Support	N,S co-functionalized carbon	2.5:1	-	-	Co-functionalized support
NSC3	Support	N,S co-functionalized carbon	2.3:1	-	-	Co-functionalized support
Pd/C	Catalyst	-	-	0.86	4.6 ± 5.3	Particle-size control
Pd/NC	Catalyst	N-functionalized	-	0.79	~1.1 ± 0.2	Activity-favored control
Pd/SC	Catalyst	S-functionalized	-	0.71	~0.7 ± 0.2	Selectivity-favored control
Pd/NSC1	Catalyst	N,S co-functionalized	2.7:1	0.83	~1	Target catalyst
Pd/NSC2	Catalyst	N,S co-functionalized	2.5:1	0.77	~1	Best-performing catalyst
Pd/NSC3	Catalyst	N,S co-functionalized	2.3:1	0.80	~1	Target catalyst

## Data Availability

The original contributions presented in this study are included in the article/Supplementary Materials. Further inquiries can be directed to the corresponding authors.

## Supplementary Materials

The following supporting information can be downloaded at https://www.mdpi.com/article/10.3390/ma19081594/s1, Figure S1: The energy-dispersive X-ray spectrometry (EDS) elementary mapping images of the NSC1 (a–d), NSC2 (e–h), and NSC3 (i–l) supports; Figure S2: Low-magnification HAADF-STEM images of the Pd/SC (a) and Pd/NC (b); Figure S3: The energy-dispersive X-ray spectrometry (EDS) elementary mapping images of the Pd/NSC1 (a–e), Pd/NSC2 (f–j), and Pd/NSC3 (k–o); Figure S4: Pd NPs that were prepared on pure carbon support (Pd/C). (a,b) Bright-field TEM and (c,d) dark-field TEM images of the Pd/C. (e–g) EDS elementary mapping images of the Pd/C; Figure S5: XRD patterns of the Pd/C. The vertical lines represent the standard peaks of the Pd (PDF no. 46-1043); Figure S6: H_2_O_2_ selectivity curves of Pd/NC and Pd/C, testing in a neutral electrolyte (0.2 M phosphate-buffered saline, PBS, pH ≈ 7); Figure S7: Stability test over the Pd/NSC2 catalysts for 12 h at 0.5 V in a neutral electrolyte (0.2 M phosphate-buffered saline, PBS, pH ≈ 7); Figure S8: Tafel plots derived from ORR polarization curves; Figure S9: Correlation between the Pd 3d5/2 binding energy and (a) H_2_O_2_ selectivity and (b) kinetic current density at 0.45 V vs. RHE for Pd/NC, Pd/SC, and Pd/NSC catalysts.
