# Supplementary material for: Support Engineering Strategy to Tackle the Trade-Off Between Catalytic Reactivity and H2O2 Selectivity in Electro-Oxygen Reduction
Source: Materials (Basel). 2026 Apr 15;19(8):1594. doi: 10.3390/ma19081594 (PMC13118052; doi:10.3390/ma19081594)
Supplement: Supplementary file 1 [file materials-19-01594-s001.zip › materials-4200507-supplementary.pdf]

Supplementary Materials

Support Engineering Strategy to Tackle the Trade-Off Between Catalytic Reactivity and H_2O_2 Selectivity in Electro-Oxygen Reduction

Zetao Song [1,†], Shuai Ran [1,†], Zengjian Cai [1,2,*], Yue Zhao [1], Xiaobing Yang [1], Zhe Wang [2], Guodong Sun [2],
Yanan Cao [2] and Li Li [1,*]

[1] State Key Laboratory of Chemistry for NBC Hazards Protection, Beijing 102205, China
[2] School of Chemical Sciences, University of Chinese Academy of Sciences, Beijing 100049, China
* Correspondence: caizengjian19@mails.ucas.ac.cn (Z.C.); lily97@buaa.edu.cn (L.L.)
† These authors contributed equally to this work.

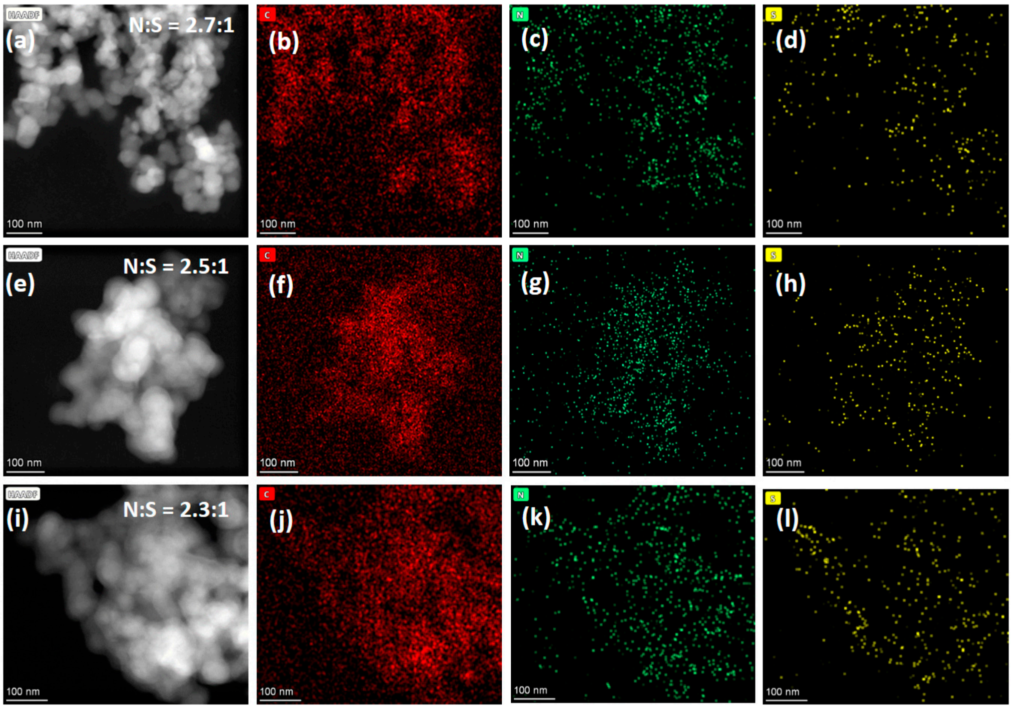

Figure S1. The energy-dispersive X-ray spectrometry (EDS) elementary mapping images of the NSC1 (a-d), NSC2 (e-h), and NSC3 (i-l) supports.

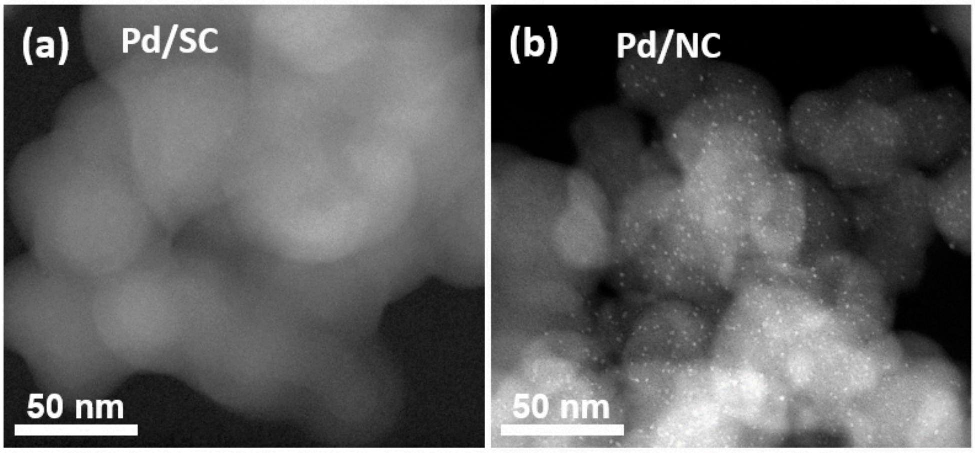

Figure S2. Low-magnification HAADF-STEM images of the Pd/SC (a) and Pd/NC (b), respectively.

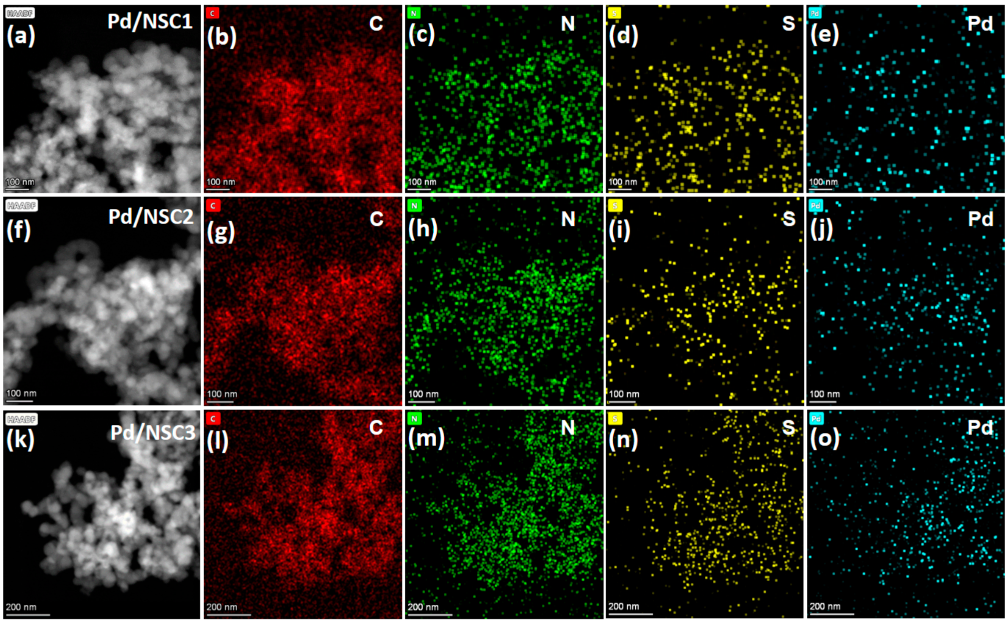

Figure S3. The energy-dispersive X-ray spectrometry (EDS) elementary mapping images of the Pd/NSC1 (a-e), Pd/NSC2 (f-j), and Pd/NSC3 (k-o).

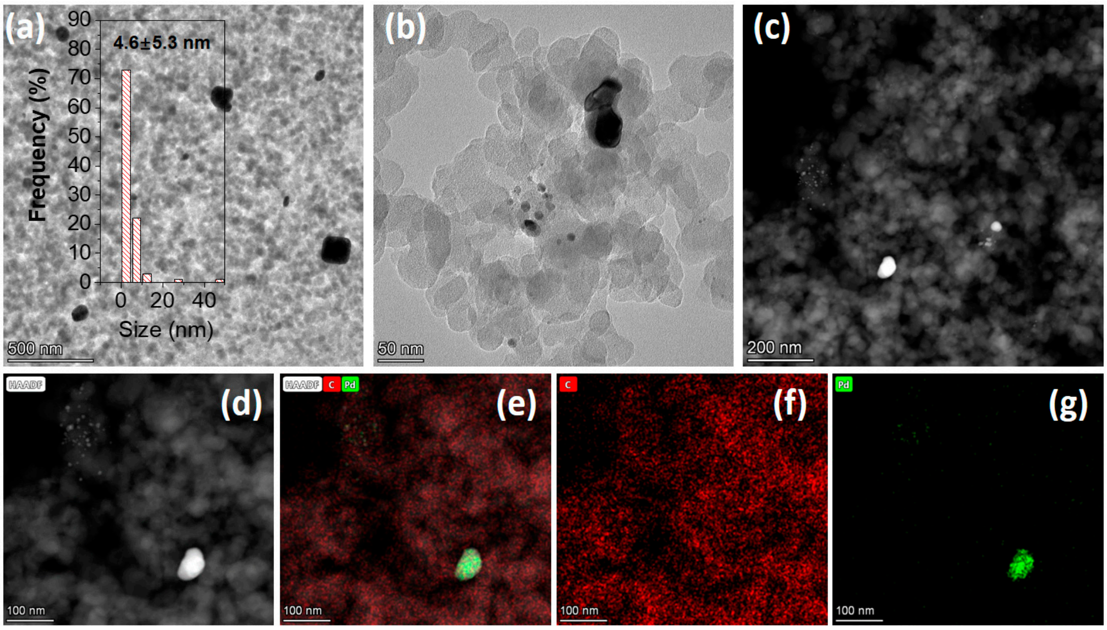

Figure S4. Pd NPs that were prepared on pure carbon support (Pd/C). (a,b) Bright-field TEM and (c,d) dark-field TEM images of the Pd/C. (e)-(g) EDS elementary mapping images of the Pd/C.

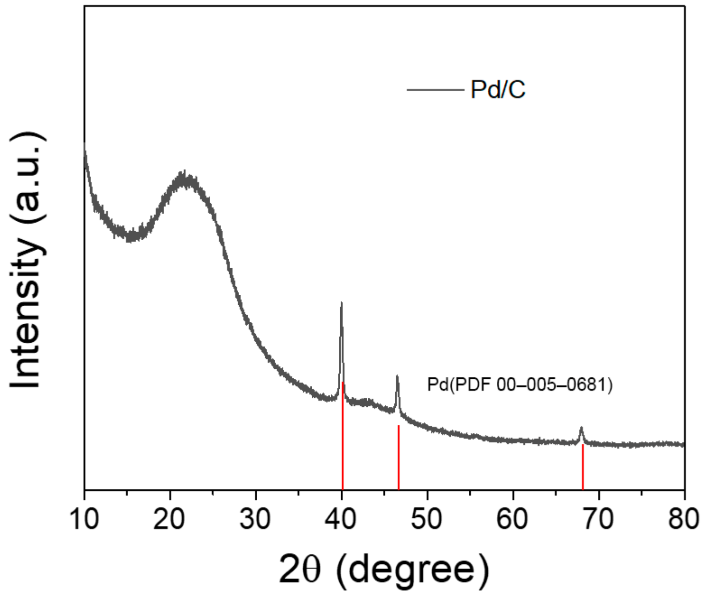

Figure S5. XRD patterns of the Pd/C. The vertical lines represent for the standard peaks of the Pd (PDF no. 46-1043).

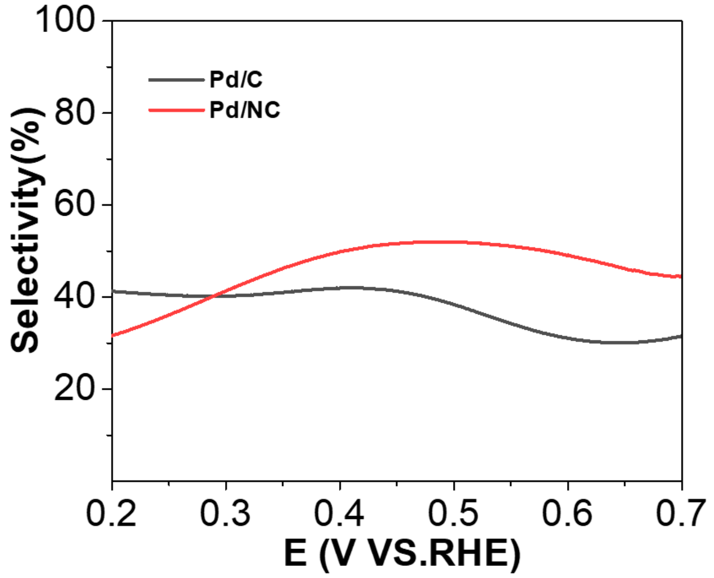

Figure S6. H_2O_2 selectivity curves of the Pd/NC and Pd/C, respectively, testing in a neutral electrolyte (0.2 M phosphate-buffered saline, PBS, pH ≈7).

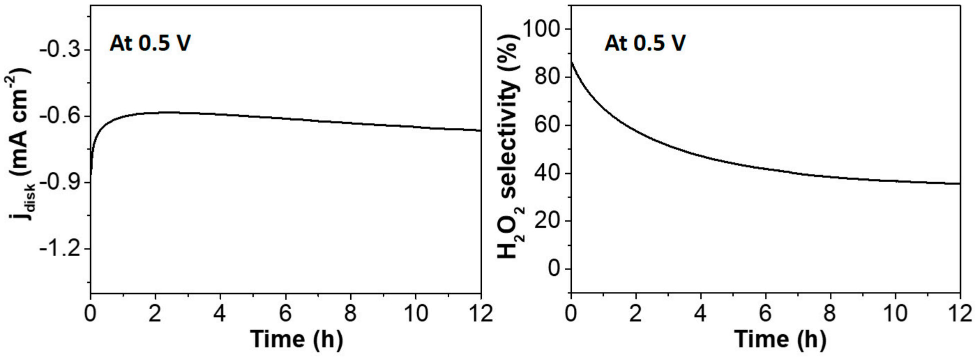

Figure S7. Stability test over the Pd/NSC2 catalysts for 12 hours at 0.5 V in a neutral electrolyte (0.2 M phosphate-buffered saline, PBS, pH≈7).

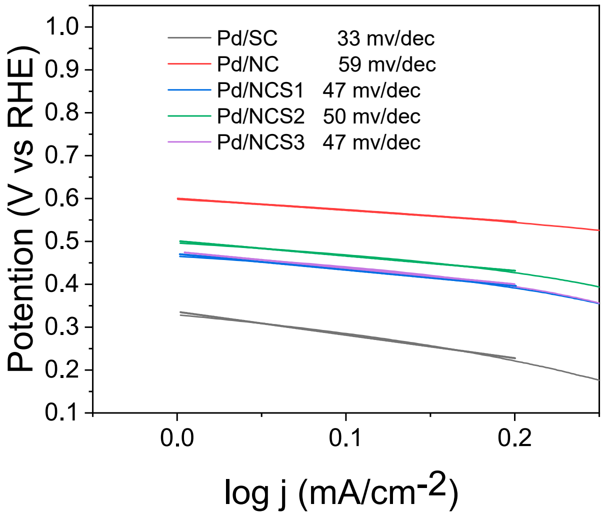

Figure S8. Tafel plots derived from ORR polarization curves.

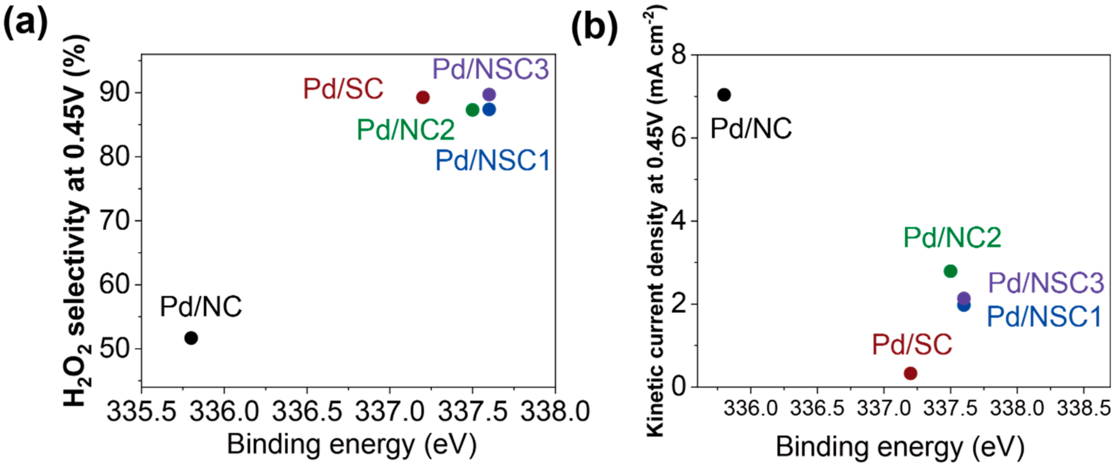

Figure S9. Correlation between the Pd $3d_{5/2}$ binding energy and (a) H_2O_2 selectivity and (b) kinetic current density at 0.45 V vs. RHE for Pd/NC, Pd/SC, and Pd/NSC catalysts.
